# A multi-dimensional view on the etiology of Parkinson’s disease

**DOI:** 10.1038/s41531-025-01150-5

**Published:** 2025-10-14

**Authors:** Rita Bernhardt, Julia Schulze-Hentrich

**Affiliations:** 1 Institut für Biochemie, Fachbereich Biologie, Naturwissenschaftlich-Technische Fakultät, Universität des Saarlandes, Saarbrücken, Germany; 2 Institut für Genetik, Fachbereich Biologie, Naturwissenschaftlich-Technische Fakultät, Universität des Saarlandes, Saarbrücken, Germany

**Keywords:** Biochemistry, Neuroscience, Diseases

## Abstract

Parkinson’s disease (PD) has been proposed to be a predominantly genetic versus a mainly environmental disease. We suggest to consider PD rather as a disease caused by multi-dimensional factors, including genetic and environmental, but also epigenetic and metabolic effects. This view is supported by very recently published data on the role of cytochromes P450 (P450s) as a model for a participation of different metabolic pathways to PD. P450s are involved in a broad variety of physiological reactions in the human body and, in addition, play a fundamental role in the detoxification of environmental compounds. Therefore, the majority of PD cases is likely based on a complex interplay between disturbances in genes and the corresponding proteins, which catalyze various metabolic pathways, as well as epigenetic, physiological and environmental effects contributing to PD symptoms. In this context, PD can be caused by one gene mutation (in rare cases), by a combination of variants in different genes, and by an association of environmental factors (e.g. pesticides) with such variants. Characterization of these factors in each individual patient opens up new avenues for a personalized therapy.

## Background and existing conceptual models for PD etiology

Parkinson’s disease (PD) is the fastest growing neurodegenerative disease in terms of prevalence with an increase to approximately 17.5 million people expected world-wide until 2040^1^. This poses a significant clinical challenge as well as a growing social concern. PD is characterized by progressive degeneration of the *Substantia nigra pars compacta* leading to a loss of dopaminergic neurons.

It is generally accepted that there are two main forms of PD: a familial and a sporadic one. Only 10–15% of all patients and about 25% of early-onset patients have a family history (first degree relatives) of PD, while the majority of PD cases is caused by largely unknown reasons^2–4^. Since the discovery of a gene mutation (Ala53Thr change in the alpha-synuclein protein encoded by SNCA) in familial forms of PD^5^, much attention has been paid to the investigation of genes involved in the genetic predisposition of PD such as *SNCA*, *LRRK2*, *GBA1* with tremendous progress during the past 30 years to better understand this disease. However, a direct causal link between these changes and the development of Parkinson’s disease is mostly still missing^6^. Moreover, when considering patients with a familial form of PD caused by mutation in one of the 7 well established PD genes (*SNCA*, *LRRK2*, *GBA1*, *PRKN*, *PINK1*, *PARK7* and *VPS35*)^7^, the penetrance of the disease is not always high and varies strongly across different populations and ethnicities^8^. While in a few cases, e.g., gene triplication of *SNCA*, full (100%) penetrance is observed, other cases show much lower penetrance^9^ indicating that a genetic predisposition is typically not sufficient to trigger the disease. The observed missing penetrance may be due to:

(i) metabolic counter-regulations to overcome the effect of the mutations,
(ii) environmentally-induced epigenetic changes in addition to a genetic predisposition,
(iii) environmental conditions driving the symptoms of the predisposed individual,
(iv) additional SNPs in other genes to manifest the disease.

Possible counter-regulations and compensations are very complex. It can be expected that depending on the underlying genetic predisposition various pathways are affected and create a counter-balancing metabolic change. Thereby a mutation in the LRRK2 gene and protein will cause different metabolic changes than a mutation in GBA1. In the context of epigenetic changes, physiological parameters of the patient (affected by nutrition, exercise, possible drug treatment against chronic diseases etc.) as well as gene-environment interactions have to be taken into account. PD animal models studying the impact of environmental influences such as high-fat diet or exercise on gene expression and epigenetic changes underline this assumption^10–12^. Human cohorts require much larger data sets, but it was shown that genetic variation and pesticide exposure influence blood DNA methylation signatures suggesting genotype, and to a lesser degree, genotype-exposure interactions contributing to variations in PD-associated DNA methylation^13^. Furthermore, investigating the association between a genetic predisposition for PD and the effect of environmental factors, a recent study clearly demonstrated that carriers of a mutation in *GBA1* have a higher risk to develop symptoms of PD upon pesticide exposure^14^. This is consistent with studies showing that 10 of the 39 pesticides linked to PD were found to be neurotoxic^15^. A growing number of studies support the effect of environmental factors on the development of PD, for both familial and sporadic forms. A prominent example is the association between the neurotoxin 1-methyl-4-phenyl-1,2,3,6-tetrahydropyridine (MPTP) and PD caused by the occurrence of so-called poor metabolizers (PMs) which have mutations in one of the P450s, CYP2D6, leading to decreased degradation of the toxin^16^. Furthermore, epidemiological data indicate a convincing association between the application of pesticides in agriculture and the occurrence of PD^17,18^. Although the mechanisms remain to be elucidated, it is plausible that, as in other cases, SNPs in genes encoding enzymes for toxic compound degradation—particularly those in the cytochrome P450 family, which are key in metabolizing exogenous substances—may play an important role, as demonstrated for CYP2D6^19^. Based on this observation and on recently published data on the association of SNPs in cytochromes P450 with PD^20,21^ (see below), we would like to demonstrate that both forms of PD, familial and sporadic, display two faces of the same medal and that multi-dimensional effects need to be taken into account to understand and treat PD.

## Cytochrome P450 enzymes as emerging contributors to the etiology of PD

Based on the contribution of P450s to the degradation of toxic compounds and the derived associations with PD, the question arose whether other members of this enzyme family would also be of importance for the etiology of PD. P450s are monooxygenases which are involved in a plethora of different reactions in the human body. They are responsible for the biotransformation of drugs, the conversion and degradation of toxic compounds, the biosynthesis of steroid hormones, the metabolism of fatty acids, eicosanoids, vitamins, sterols and for other yet unknown reactions^19^. This way P450s can regulate and compensate a plethora of changes caused by variants and mutations in different genes. Moreover, P450s are valuable model systems to study the influence of various noxes and pathways on the development of PD. Hence, in a recent study, we investigated their possible association with PD. Using the PPMI data base, it has been demonstrated by systematically analyzing SNPs in the genomes of all 57 human P450s and their three redox partners that 26 of these enzymes display significant over-representation of variants (at least 5-fold and up to more than 10-fold, based on calculated odds ratios, mostly found in regulatory regions) in PD patients compared with healthy controls^20^. Interestingly, P450s involved in the degradation of toxic compounds were among those showing a significant over- or under-representation of variants in PD patients compared with controls, in accordance with the role of toxic compounds as important player in the etiology of PD (see above). In addition, P450s catalyzing cholesterol degradation in the brain and participating in the metabolism of eicosanoids as well as CYP20A1-catalyzed reactions were shown to be associated with the occurrence of PD^20^. The prominent role of P450s in the etiology of PD becomes even more evident when looking at patients described with mutations in known PD genes (GPD) causing familial PD and those which display such mutations, but are without symptoms (GUN), e.g. showing low penetrance. In these analyses it turned out that the shift from a symptom-free condition to PD (GUN → GPD) occurs, when in addition to a known genetic predisposition (e.g., mutation in LRRK2 or GBA1) a variant is present in one of the P450 genes^21^. Three metabolic pathways were especially involved: (i) vitamin A and D metabolism, (ii) degradation of cholesterol and (iii) fatty acid/eicosanoid metabolism.

Moreover, an association of CYP1B1 involved in estrogen and vitamin A metabolism with PD was very recently described^22^. It is notable that the frequency of variants in some of the P450s is in the same order of magnitude than variants observed in *GBA1*, one of the well-known PD genes^23^. Thereby it is of special interest that the associated SNPs were not only found in GPD patients, but also in GUN and healthy controls. The same observation was made for *GBA1*, where mutations were not only found in PD patients, but also in healthy controls (10.1% vs. 3.8% and 15% vs. 3%, respectively) demonstrating an incomplete penetrance^24,25^. This shows that predisposition to PD in several cases needs additional variants in other genes such as P450s to become symptomatic. Collectively, these results further indicate that P450s play a significant role in gene-environment as well as gene-gene interactions associated with the occurrence of PD. Due to their versatility to catalyze important metabolic pathways they may also be involved in counter-reactions in GPD patients. Taken together, this data demonstrates that P450s are new players in the pathogenesis of PD. Significant over- or under-representations of variants in several P450 genes in PD patients vs. healthy controls clearly indicate their role in PD pathogenesis. The data also suggest that a single associated SNP in one of the P450s is not sufficient to lead to PD symptoms, since the variants also occur in healthy controls. Thus, SNPs in P450s are contributing, together with SNPs in other genes (e.g., in LRRK2) or with exposure to toxic compounds, to gene-gene or gene-environment interactions as pathogenic factors for PD.

## What are the consequences of these observations?

The discussed results indicate that

**Fig. 1:**
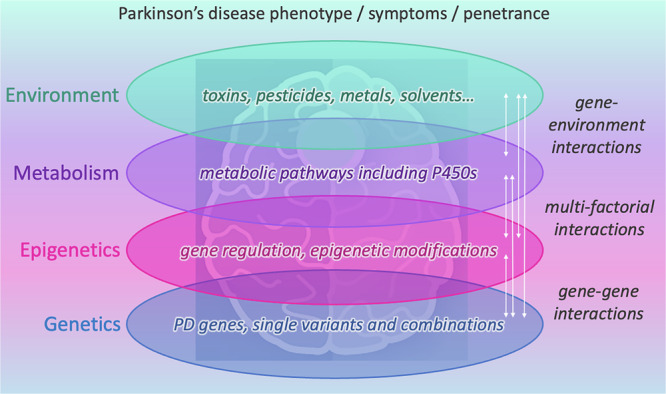
The multi-dimensional view on Parkinson’s disease (PD).

(i) PD is not a predominantly genetic or environmental disease, but rather a multifactorial disorder with a complex interplay of multiple dimensions (Fig. 1).

This conclusion is fully in line with the gut-driven model of Braak and the dual-hit hypotheses^26^. Moreover, it also encompasses the complex interaction between inflammation, genetic predisposition, environmental factors (infections, pesticides) as well as aging processes^27^. Although in rare cases pathogenic variants of PD genes may lead to full penetrance of the disease and to an autosomal dominant PD, the overwhelming majority of cases seems to be based on variations in different genes, in addition to factors such as environmental exposures that shape the epigenome. This is supported by large-scale case-control GWAS, which lead to the identification of 90 common genetic risk variants (SNPs) that are associated with PD^4^. Moreover, different and new variants of PD gene mutations seem to occur in different populations^9,28^.

(ii) Novel players involved in the pathogenesis of PD are P450s, which participate in many important pathways in the human body and should be taken into account when investigating changes in the genomes of PD patients.

Based on the results with P450s one can assume that various combinations between different PD genes with associated SNPs in different P450s lead to symptoms of the disease (e.g., mutation in LRRK2 or GBA1 plus an associated SNP in CYP27A1 and/or CYP26B1 etc.). Due to the multitude of reactions catalyzed by P450s various metabolic pathways may affect PD etiology suggesting a novel and more complex concept for the etiology of PD. It can be extrapolated that possibly also combinations of mutations/SNPs in identified PD genes or variants in different P450s with SNPs in other physiologically important genes will cause PD. This means that two or more hits are necessary to cause PD and that a plethora of combinations will lead to the disease when considering different patients (Fig. 1).

(iii) Finally, also epigenetic differences may play a pivotal role in the pathogenesis of PD.

It is becoming increasingly clear that epigenetic changes are key mechanisms by which environmental factors act upon gene regulation in disease-relevant cell types^29^.

Altogether, this on the one hand can explain the variety of phenotypes of this disease, but on the other hand may impair the development of a universal causative treatment. At the same time, it opens ways for personalized causative treatments, which at least may slow down the progression of the disease.

## Novel and future possibilities to causatively treat PD

As the etiology of PD is still highly debated, a causative treatment is yet not possible but urgently needed. Even patients with a clear monogenic cause do not receive the best treatment for their specific mutation, emphasizing the need for a systematic analysis of treatment options and outcomes^30^. To date, there are several different approaches trying to understand and to treat this disease. Based on data that α-synuclein aggregation plays a key role in the manifestation of PD, trials to treat PD patients with antibodies against this protein were started. Unfortunately, they were not successful for a broader application^31^.

This leads to the question whether a general causative treatment of PD is possible at all. Even transplantation of stem cells with functional production of dopamine may not be successful over time as long as the origin of the death of dopaminergic neurons has not been eliminated in patients. Therefore, we would like to draw the attention to a broader look at the etiology of the disease and inferable novel approaches for treatment.

A promising novel approach to such a personalized therapy is the application of small inhibitors of the kinase LRRK2^32^. This treatment should be useful for patients suffering from a gain-of-function mutation of the kinase LRRK2. The observation that various P450s are involved in the etiology of PD^20^ indicates that different physiological pathways catalyzed by P450s (e.g., metabolism of eicosanoids or vitamin D; degradation of brain cholesterol) as well as gene-environment interactions (affected by P450s degrading toxic compounds) are changed in PD. Eicosanoids play a prominent role in the immune system and in inflammation^26,27^ and exposure to pesticides and pollutants has been correlated with the occurrence of PD^14,15,17,18^, confirming the data found in the CYPome study^20^. Thus, it is conceivable that e.g., in the case of defects in vitamin D metabolism, supplementation of the corresponding patients with vitamin D or inhibition of vitamin D degradation can improve their conditions. Since P450s are well-investigated targets for drug treatment and intervention^33^ (inhibitors of aromatase are e.g., widely used in the treatment of breast cancer), known inhibitors for P450s will be valuable tools and drug lead compounds to develop a personalized treatment for PD patients. However, since several hits may cause PD (as discussed before), multiple interventions based on changed functions of the various participating genes/proteins will be necessary to improve treatment outcome. As mentioned above, whole-genome sequencing, preferably combined with the application of AI, instead of sequencing only known PD loci is necessary in scientific studies to disclose novel SNPs in so far not investigated genes and to extract combinations of variants (SNPs) in different genes of PD patients linked to PD symptoms. At the same time limited sequencing of known positions should be performed in clinical studies to look for familial PD and provide corresponding human genetic counseling^34^. Although whole-genome sequencing may be cost-intensive on a first glance, it will pay off due to the possibility of a more targeted and personalized treatment. Examples of a possible personalized treatment based on SNPs in various cholesterol-degrading P450s have been discussed in Petkova-Kirova et al.^23^. Moreover, examples are available for other neurodegenerative diseases indicating a successful treatment of patients based on the analysis of SNPs in cytochromes P450. An infantile 7α-hydroxylase deficiency due to CYP7B1 mutations has been successfully treated with oral chenodeoxycholic acid^35^. On the other hand, voriconazole was demonstrated to have implications for a personalized treatment of PD patients with a dysregulated cholesterol metabolism^36^. In addition, it has been demonstrated that mutations in CYP46A1 are connected to Alzheimer’s disease and might be treatable with efavirenz^37^ which is now in a phase III study. This also might be applicable to PD patients suffering from CYP46A1-based decrease in brain cholesterol degradation.

Sequencing whole genomes, ideally in combination with an epigenome, can, in addition, help to find new approaches not only for a personalized therapy, but also for the prevention of PD. Thus, sequencing of P450 genes may be helpful for individuals working in industries or agriculture with high exposure to pesticides, since a clear association between variations in P450s involved in the conversion/degradation of xenobiotics/toxic compounds and PD has been observed^20^. Genomes of individuals with occupational toxin exposure could be sequenced, and if PD-associated variants are identified in toxin-metabolizing P450 genes, those individuals could be advised to take specific precautions to ensure better protection from toxic substances.

## Conclusions

Taken together, we would like to suggest a new paradigm for PD, which combines the very recently expressed opinions that PD is predominantly an environmental^38^ or a genetic^9^ disease (including also gut-brain interactions and inflammatory processes^26,27^) and draw the attention to the role of metabolic changes and to the multi-dimensional character of this disease, where gene-gene and gene-environment interaction may be combined with SNPs in other genes, epigenetic changes etc. Thus, PD seems to be caused by variations in the function of many different genes with a multitude of combinations among them as well as by a multitude of various epigenetic and environmental effects. P450s, where associations between SNPs and PD have been recently discovered as novel players in the pathogenesis of PD^20^, are models for such potential interconnections between different gene variants as well as between genes, metabolism, environment and epigenetics^23^. P450s are involved in many different pathways in the human body^19^ and may be crucial to connect various factors responsible for the etiology of PD. Therefore, they require more attention as currently dedicated and more studies are necessary to derive functional consequences of the observed SNPs and relate them to the pathogenesis of this disease. It has been demonstrated that SNPs in one of the P450 genes can drive individuals with a genetic predisposition without symptoms to patients with symptoms^21^. In a more general view, this shows that the occurrence of a second (and maybe even third) hit seems to be necessary to lead to PD symptoms in predisposed individuals. Finally, interactions between environmental noxes and variations in a P450 gene underline the crucial effect of environmental factors for the pathogenesis of PD. With regard to P450 variants, known inducers and inhibitors of this group of enzymes provide an example for a starting point concerning the development of personalized drug treatment. In a broader context, this means that every patient needs to be treated in a personalized and multi-faced way based on the combination of disturbances in the patient’s metabolism.

## Data Availability

No datasets were generated or analysed during the current study.
